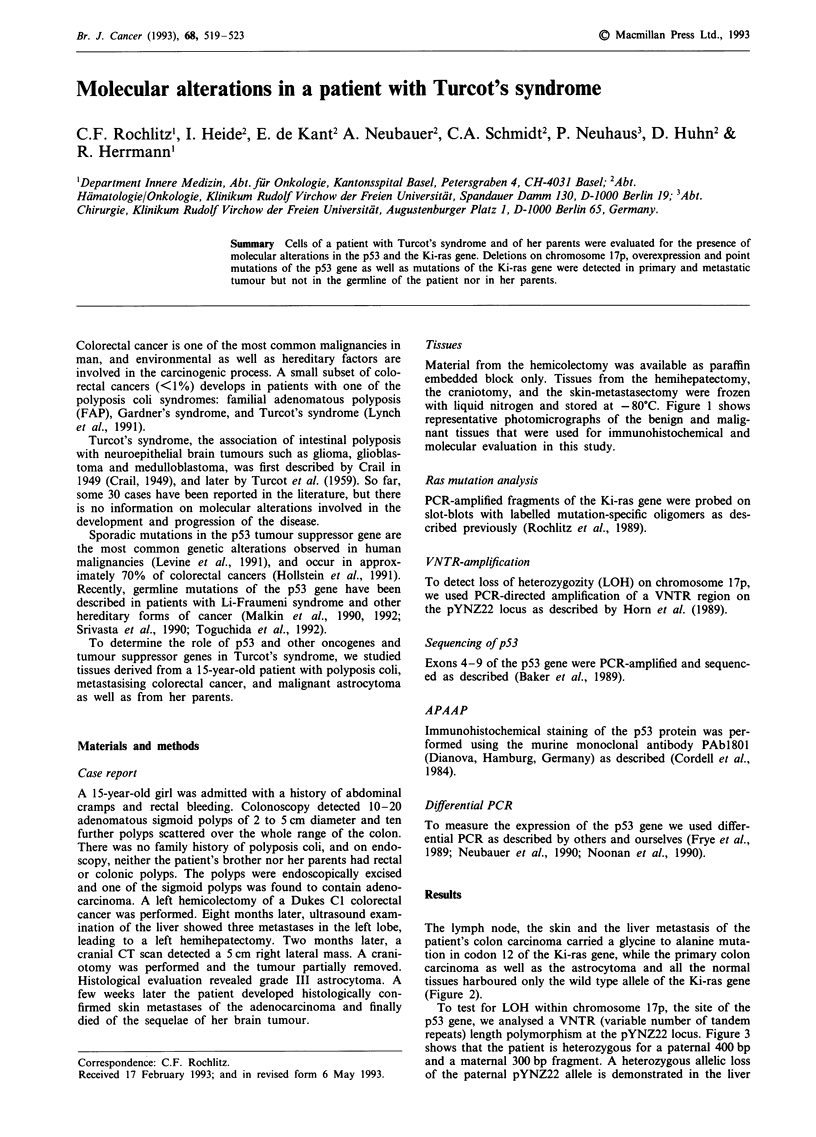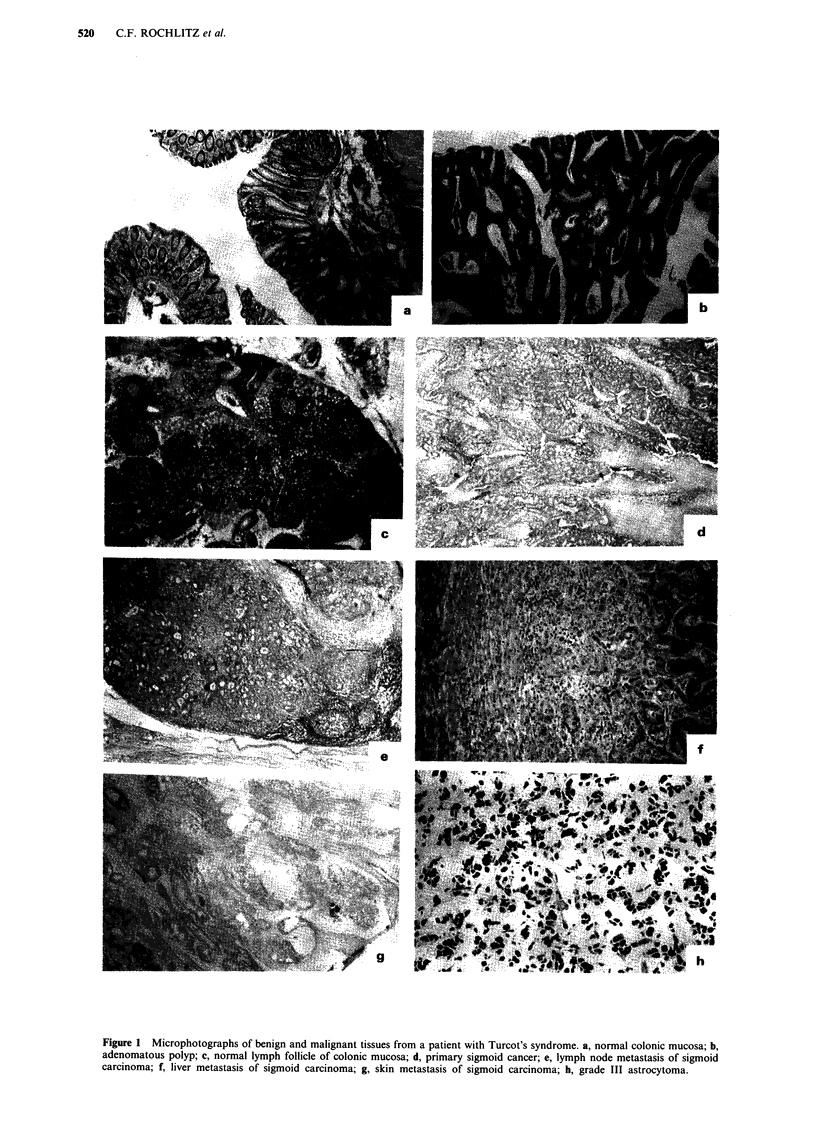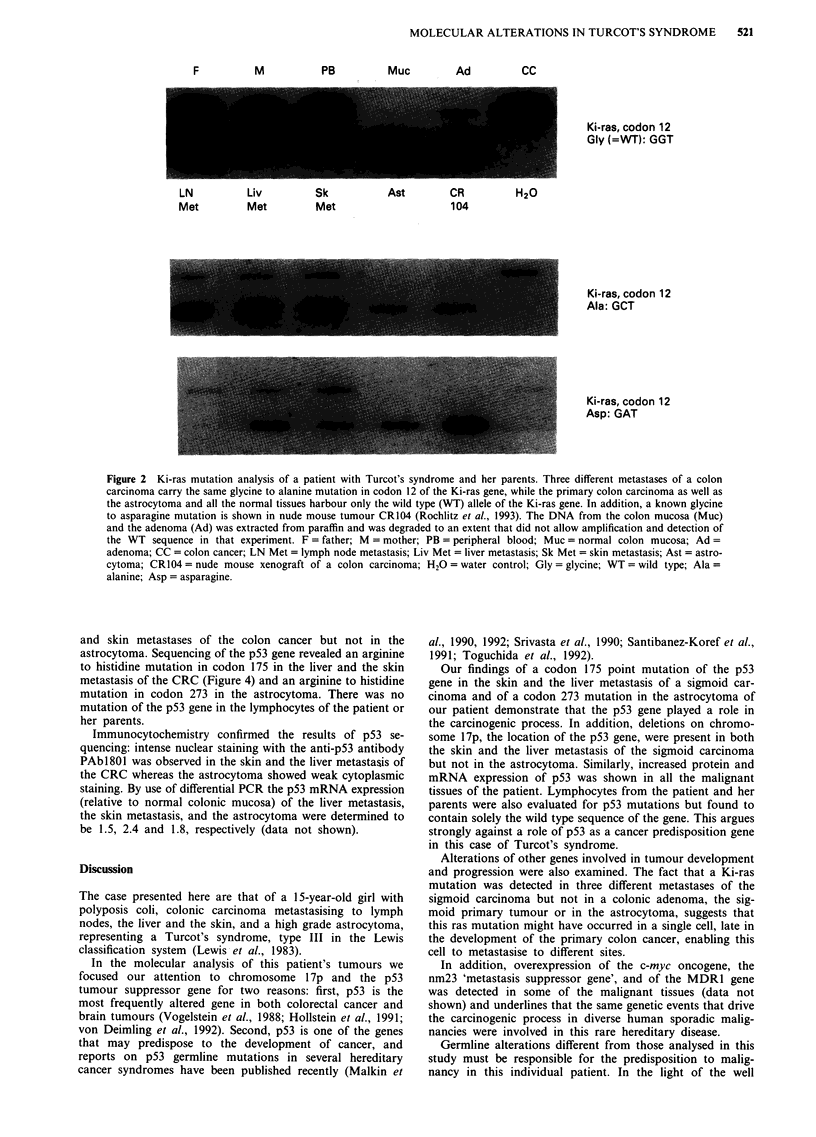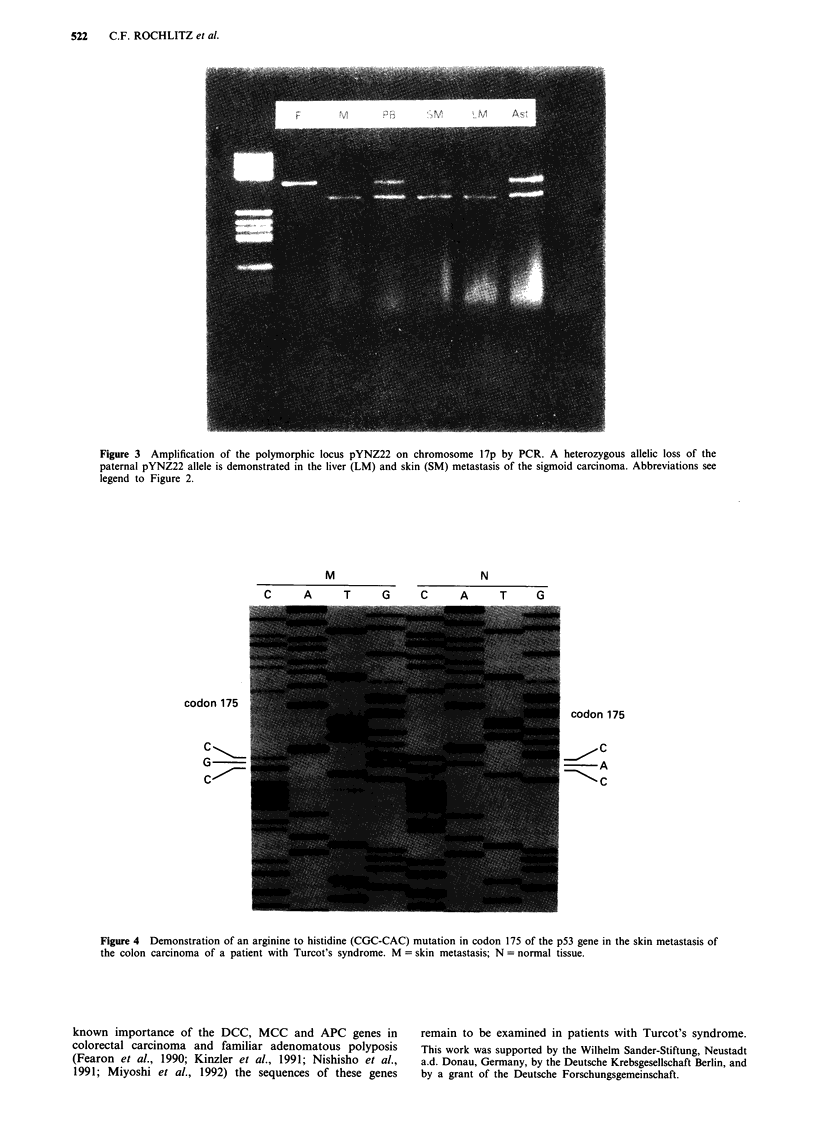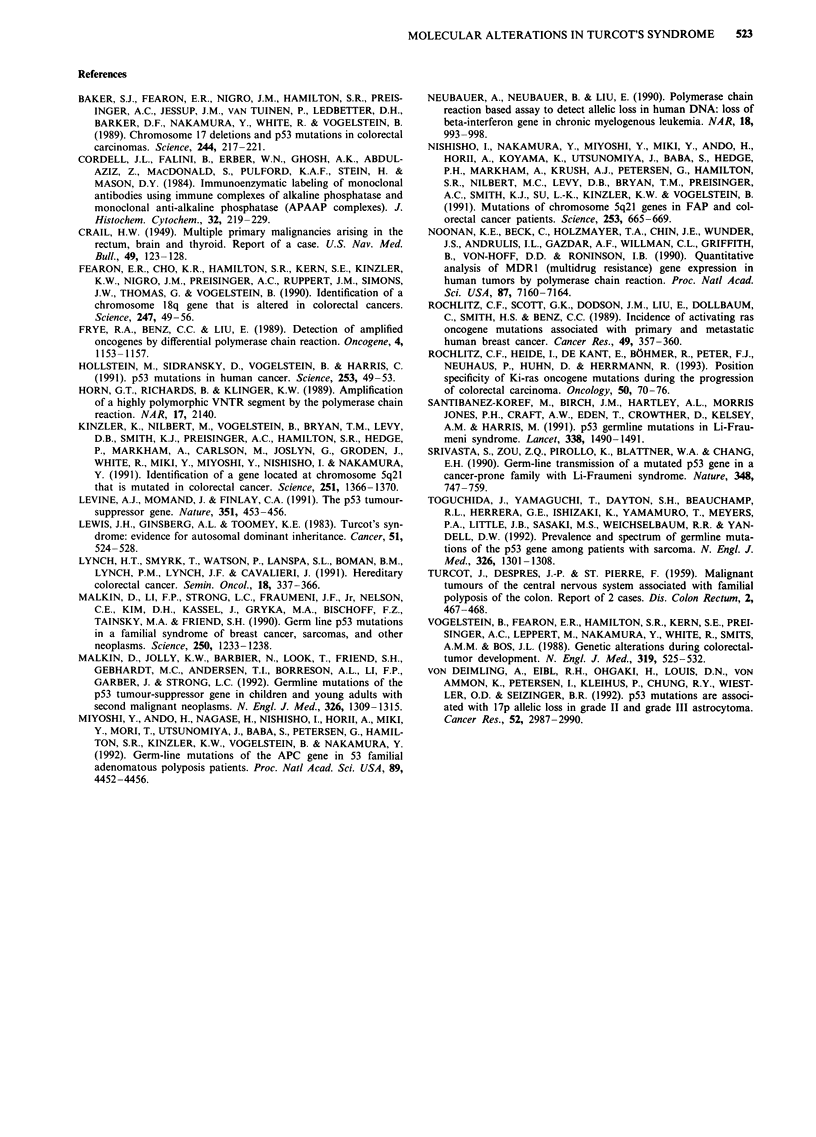# Molecular alterations in a patient with Turcot's syndrome.

**DOI:** 10.1038/bjc.1993.379

**Published:** 1993-09

**Authors:** C. F. Rochlitz, I. Heide, E. de Kant, A. Neubauer, C. A. Schmidt, P. Neuhaus, D. Huhn, R. Herrmann

**Affiliations:** Department Innere Medizin, Kantonsspital Basel.

## Abstract

**Images:**


Br. J. Cancer (1993), 68, 519–523 © Macmillan Press Ltd., 1993

### Molecular alterations in a patient with Turcot's syndrome

C.F. Rochlitz[1], I. Heide[2], E. de Kant[2] A. Neubauer[2], C.A. Schmidt[2], P. Neuhaus[3], D. Huhn[2] & R. Herrmann[1]

[1]*Department Innere Medizin, Abt. für Onkologie, Kantonsspital Basel, Petersgraben 4, CH-4031 Basel;* [2]*Abt. Hämatologie/Onkologie, Klinikum Rudolf Virchow der Freien Universität, Spandauer Damm 130, D-1000 Berlin 19;* [3]*Abt. Chirurgie, Klinikum Rudolf Virchow der Freien Universität, Augustenburger Platz 1, D-1000 Berlin 65, Germany.*

**Summary** Cells of a patient with Turcot's syndrome and of her parents were evaluated for the presence of molecular alterations in the p53 and the Ki-ras gene. Deletions on chromosome 17p, overexpression and point mutations of the p53 gene as well as mutations of the Ki-ras gene were detected in primary and metastatic tumour but not in the germline of the patient nor in her parents.

Colorectal cancer is one of the most common malignancies in man, and environmental as well as hereditary factors are involved in the carcinogenic process. A small subset of colorectal cancers (<1%) develops in patients with one of the polyposis coli syndromes: familial adenomatous polyposis (FAP), Gardner's syndrome, and Turcot's syndrome (Lynch *et al.*, 1991).

Turcot's syndrome, the association of intestinal polyposis with neuroepithelial brain tumours such as glioma, glioblastoma and medulloblastoma, was first described by Crail in 1949 (Crail, 1949), and later by Turcot *et al.* (1959). So far, some 30 cases have been reported in the literature, but there is no information on molecular alterations involved in the development and progression of the disease.

Sporadic mutations in the p53 tumour suppressor gene are the most common genetic alterations observed in human malignancies (Levine *et al.*, 1991), and occur in approximately 70% of colorectal cancers (Hollstein *et al.*, 1991). Recently, germline mutations of the p53 gene have been described in patients with Li-Fraumeni syndrome and other hereditary forms of cancer (Malkin *et al.*, 1990, 1992; Srivasta *et al.*, 1990; Toguchida *et al.*, 1992).

To determine the role of p53 and other oncogenes and tumour suppressor genes in Turcot's syndrome, we studied tissues derived from a 15-year-old patient with polyposis coli, metastasising colorectal cancer, and malignant astrocytoma as well as from her parents.

### Materials and methods

### *Case report*

A 15-year-old girl was admitted with a history of abdominal cramps and rectal bleeding. Colonoscopy detected 10–20 adenomatous sigmoid polyps of 2 to 5 cm diameter and ten further polyps scattered over the whole range of the colon. There was no family history of polyposis coli, and on endoscopy, neither the patient's brother nor her parents had rectal or colonic polyps. The polyps were endoscopically excised and one of the sigmoid polyps was found to contain adenocarcinoma. A left hemicolectomy of a Dukes C1 colorectal cancer was performed. Eight months later, ultrasound examination of the liver showed three metastases in the left lobe, leading to a left hemihepatectomy. Two months later, a cranial CT scan detected a 5 cm right lateral mass. A craniotomy was performed and the tumour partially removed. Histological evaluation revealed grade III astrocytoma. A few weeks later the patient developed histologically confirmed skin metastases of the adenocarcinoma and finally died of the sequelae of her brain tumour.

Correspondence: C.F. Rochlitz.
Received 17 February 1993; and in revised form 6 May 1993.

### *Tissues*

Material from the hemicolectomy was available as paraffin embedded block only. Tissues from the hemihepatectomy, the craniotomy, and the skin-metastasectomy were frozen with liquid nitrogen and stored at −80°C. Figure 1 shows representative photomicrographs of the benign and malignant tissues that were used for immunohistochemical and molecular evaluation in this study.

### *Ras mutation analysis*

PCR-amplified fragments of the Ki-ras gene were probed on slot-blots with labelled mutation-specific oligomers as described previously (Rochlitz *et al.*, 1989).

### *VNTR-amplification*

To detect loss of heterozygozity (LOH) on chromosome 17p, we used PCR-directed amplification of a VNTR region on the pYNZ22 locus as described by Horn *et al.* (1989).

### *Sequencing of p53*

Exons 4–9 of the p53 gene were PCR-amplified and sequenced as described (Baker *et al.*, 1989).

### *APAAP*

Immunohistochemical staining of the p53 protein was performed using the murine monoclonal antibody PAb1801 (Dianova, Hamburg, Germany) as described (Cordell *et al.*, 1984).

### *Differential PCR*

To measure the expression of the p53 gene we used differential PCR as described by others and ourselves (Frye *et al.*, 1989; Neubauer *et al.*, 1990; Noonan *et al.*, 1990).

### Results

The lymph node, the skin and the liver metastasis of the patient's colon carcinoma carried a glycine to alanine mutation in codon 12 of the Ki-ras gene, while the primary colon carcinoma as well as the astrocytoma and all the normal tissues harboured only the wild type allele of the Ki-ras gene (Figure 2).

To test for LOH within chromosome 17p, the site of the p53 gene, we analysed a VNTR (variable number of tandem repeats) length polymorphism at the pYNZ22 locus. Figure 3 shows that the patient is heterozygous for a paternal 400 bp and a maternal 300 bp fragment. A heterozygous allelic loss of the paternal pYNZ22 allele is demonstrated in the liver

**Figure 1** Microphotographs of benign and malignant tissues from a patient with Turcot's syndrome. **a**, normal colonic mucosa; **b**, adenomatous polyp; **c**, normal lymph follicle of colonic mucosa; **d**, primary sigmoid cancer; **e**, lymph node metastasis of sigmoid carcinoma; **f**, liver metastasis of sigmoid carcinoma; **g**, skin metastasis of sigmoid carcinoma; **h**, grade III astrocytoma.

**Figure 2** Ki-ras mutation analysis of a patient with Turcot's syndrome and her parents. Three different metastases of a colon carcinoma carry the same glycine to alanine mutation in codon 12 of the Ki-ras gene, while the primary colon carcinoma as well as the astrocytoma and all the normal tissues harbour only the wild type (WT) allele of the Ki-ras gene. In addition, a known glycine to asparagine mutation is shown in nude mouse tumour CR104 (Rochlitz *et al*., 1993). The DNA from the colon mucosa (Muc) and the adenoma (Ad) was extracted from paraffin and was degraded to an extent that did not allow amplification and detection of the WT sequence in that experiment. F = father; M = mother; PB = peripheral blood; Muc = normal colon mucosa; Ad = adenoma; CC = colon cancer; LN Met = lymph node metastasis; Liv Met = liver metastasis; Sk Met = skin metastasis; Ast = astrocytoma; CR104 = nude mouse xenograft of a colon carcinoma; $H_2O$ = water control; Gly = glycine; WT = wild type; Ala = alanine; Asp = asparagine.

and skin metastases of the colon cancer but not in the astrocytoma. Sequencing of the p53 gene revealed an arginine to histidine mutation in codon 175 in the liver and the skin metastasis of the CRC (Figure 4) and an arginine to histidine mutation in codon 273 in the astrocytoma. There was no mutation of the p53 gene in the lymphocytes of the patient or her parents.

Immunocytochemistry confirmed the results of p53 sequencing: intense nuclear staining with the anti-p53 antibody PAb1801 was observed in the skin and the liver metastasis of the CRC whereas the astrocytoma showed weak cytoplasmic staining. By use of differential PCR the p53 mRNA expression (relative to normal colonic mucosa) of the liver metastasis, the skin metastasis, and the astrocytoma were determined to be 1.5, 2.4 and 1.8, respectively (data not shown).

### Discussion

The case presented here are that of a 15-year-old girl with polyposis coli, colonic carcinoma metastasising to lymph nodes, the liver and the skin, and a high grade astrocytoma, representing a Turcot's syndrome, type III in the Lewis classification system (Lewis *et al*., 1983).

In the molecular analysis of this patient's tumours we focused our attention to chromosome 17p and the p53 tumour suppressor gene for two reasons: first, p53 is the most frequently altered gene in both colorectal cancer and brain tumours (Vogelstein *et al*., 1988; Hollstein *et al*., 1991; von Deimling *et al*., 1992). Second, p53 is one of the genes that may predispose to the development of cancer, and reports on p53 germline mutations in several hereditary cancer syndromes have been published recently (Malkin *et al*., 1990, 1992; Srivasta *et al*., 1990; Santibanez-Koref *et al*., 1991; Toguchida *et al*., 1992).

Our findings of a codon 175 point mutation of the p53 gene in the skin and the liver metastasis of a sigmoid carcinoma and of a codon 273 mutation in the astrocytoma of our patient demonstrate that the p53 gene played a role in the carcinogenic process. In addition, deletions on chromosome 17p, the location of the p53 gene, were present in both the skin and the liver metastasis of the sigmoid carcinoma but not in the astrocytoma. Similarly, increased protein and mRNA expression of p53 was shown in all the malignant tissues of the patient. Lymphocytes from the patient and her parents were also evaluated for p53 mutations but found to contain solely the wild type sequence of the gene. This argues strongly against a role of p53 as a cancer predisposition gene in this case of Turcot's syndrome.

Alterations of other genes involved in tumour development and progression were also examined. The fact that a Ki-ras mutation was detected in three different metastases of the sigmoid carcinoma but not in a colonic adenoma, the sigmoid primary tumour or in the astrocytoma, suggests that this ras mutation might have occurred in a single cell, late in the development of the primary colon cancer, enabling this cell to metastasise to different sites.

In addition, overexpression of the c-*myc* oncogene, the nm23 'metastasis suppressor gene', and of the MDR1 gene was detected in some of the malignant tissues (data not shown) and underlines that the same genetic events that drive the carcinogenic process in diverse human sporadic malignancies were involved in this rare hereditary disease.

Germline alterations different from those analysed in this study must be responsible for the predisposition to malignancy in this individual patient. In the light of the well

**Figure 3** Amplification of the polymorphic locus pYNZ22 on chromosome 17p by PCR. A heterozygous allelic loss of the paternal pYNZ22 allele is demonstrated in the liver (LM) and skin (SM) metastasis of the sigmoid carcinoma. Abbreviations see legend to Figure 2.

**Figure 4** Demonstration of an arginine to histidine (CGC-CAC) mutation in codon 175 of the p53 gene in the skin metastasis of the colon carcinoma of a patient with Turcot's syndrome. M = skin metastasis; N = normal tissue.

known importance of the DCC, MCC and APC genes in colorectal carcinoma and familiar adenomatous polyposis (Fearon *et al.*, 1990; Kinzler *et al.*, 1991; Nishisho *et al.*, 1991; Miyoshi *et al.*, 1992) the sequences of these genes remain to be examined in patients with Turcot's syndrome.

This work was supported by the Wilhelm Sander-Stiftung, Neustadt a.d. Donau, Germany, by the Deutsche Krebsgesellschaft Berlin, and by a grant of the Deutsche Forschungsgemeinschaft.

**References**

BAKER, S.J., FEARON, E.R., NIGRO, J.M., HAMILTON, S.R., PREISINGER, A.C., JESSUP, J.M., VAN TUINEN, P., LEDBETTER, D.H., BARKER, D.F., NAKAMURA, Y., WHITE, R. & VOGELSTEIN, B. (1989). Chromosome 17 deletions and p53 mutations in colorectal carcinomas. *Science*, **244**, 217–221.

CORDELL, J.L., FALINI, B., ERBER, W.N., GHOSH, A.K., ABDUL-AZIZ, Z., MACDONALD, S., PULFORD, K.A.F., STEIN, H. & MASON, D.Y. (1984). Immunoenzymatic labeling of monoclonal antibodies using immune complexes of alkaline phosphatase and monoclonal anti-alkaline phosphatase (APAAP complexes). *J. Histochem. Cytochem.*, **32**, 219–229.

CRAIL, H.W. (1949). Multiple primary malignancies arising in the rectum, brain and thyroid. Report of a case. *U.S. Nav. Med. Bull.*, **49**, 123–128.

FEARON, E.R., CHO, K.R., HAMILTON, S.R., KERN, S.E., KINZLER, K.W., NIGRO, J.M., PREISINGER, A.C., RUPPERT, J.M., SIMONS, J.W., THOMAS, G. & VOGELSTEIN, B. (1990). Identification of a chromosome 18q gene that is altered in colorectal cancers. *Science*, **247**, 49–56.

FRYE, R.A., BENZ, C.C. & LIU, E. (1989). Detection of amplified oncogenes by differential polymerase chain reaction. *Oncogene*, **4**, 1153–1157.

HOLLSTEIN, M., SIDRANSKY, D., VOGELSTEIN, B. & HARRIS, C. (1991). p53 mutations in human cancer. *Science*, **253**, 49–53.

HORN, G.T., RICHARDS, B. & KLINGER, K.W. (1989). Amplification of a highly polymorphic VNTR segment by the polymerase chain reaction. *NAR*, **17**, 2140.

KINZLER, K., NILBERT, M., VOGELSTEIN, B., BRYAN, T.M., LEVY, D.B., SMITH, K.J., PREISINGER, A.C., HAMILTON, S.R., HEDGE, P., MARKHAM, A., CARLSON, M., JOSLYN, G., GRODEN, J., WHITE, R., MIKI, Y., MIYOSHI, Y., NISHISHO, I. & NAKAMURA, Y. (1991). Identification of a gene located at chromosome 5q21 that is mutated in colorectal cancer. *Science*, **251**, 1366–1370.

LEVINE, A.J., MOMAND, J. & FINLAY, C.A. (1991). The p53 tumour-suppressor gene. *Nature*, **351**, 453–456.

LEWIS, J.H., GINSBERG, A.L. & TOOMEY, K.E. (1983). Turcot's syndrome: evidence for autosomal dominant inheritance. *Cancer*, **51**, 524–528.

LYNCH, H.T., SMYRK, T., WATSON, P., LANSPA, S.L., BOMAN, B.M., LYNCH, P.M., LYNCH, J.F. & CAVALIERI, J. (1991). Hereditary colorectal cancer. *Semin. Oncol.*, **18**, 337–366.

MALKIN, D., LI, F.P., STRONG, L.C., FRAUMENI, J.F., Jr, NELSON, C.E., KIM, D.H., KASSEL, J., GRYKA, M.A., BISCHOFF, F.Z., TAINSKY, M.A. & FRIEND, S.H. (1990). Germ line p53 mutations in a familial syndrome of breast cancer, sarcomas, and other neoplasms. *Science*, **250**, 1233–1238.

MALKIN, D., JOLLY, K.W., BARBIER, N., LOOK, T., FRIEND, S.H., GEBHARDT, M.C., ANDERSEN, T.I., BORRESON, A.L., LI, F.P., GARBER, J. & STRONG, L.C. (1992). Germline mutations of the p53 tumour-suppressor gene in children and young adults with second malignant neoplasms. *N. Engl. J. Med.*, **326**, 1309–1315.

MIYOSHI, Y., ANDO, H., NAGASE, H., NISHISHO, I., HORII, A., MIKI, Y., MORI, T., UTSUNOMIYA, J., BABA, S., PETERSEN, G., HAMILTON, S.R., KINZLER, K.W., VOGELSTEIN, B. & NAKAMURA, Y. (1992). Germ-line mutations of the APC gene in 53 familial adenomatous polyposis patients. *Proc. Natl Acad. Sci. USA*, **89**, 4452–4456.

NEUBAUER, A., NEUBAUER, B. & LIU, E. (1990). Polymerase chain reaction based assay to detect allelic loss in human DNA: loss of beta-interferon gene in chronic myelogenous leukemia. *NAR*, **18**, 993–998.

NISHISHO, I., NAKAMURA, Y., MIYOSHI, Y., MIKI, Y., ANDO, H., HORII, A., KOYAMA, K., UTSUNOMIYA, J., BABA, S., HEDGE, P.H., MARKHAM, A., KRUSH, A.J., PETERSEN, G., HAMILTON, S.R., NILBERT, M.C., LEVY, D.B., BRYAN, T.M., PREISINGER, A.C., SMITH, K.J., SU, L.-K., KINZLER, K.W. & VOGELSTEIN, B. (1991). Mutations of chromosome 5q21 genes in FAP and colorectal cancer patients. *Science*, **253**, 665–669.

NOONAN, K.E., BECK, C., HOLZMAYER, T.A., CHIN, J.E., WUNDER, J.S., ANDRULIS, I.L., GAZDAR, A.F., WILLMAN, C.L., GRIFFITH, B., VON-HOFF, D.D. & RONINSON, I.B. (1990). Quantitative analysis of MDR1 (multidrug resistance) gene expression in human tumors by polymerase chain reaction. *Proc. Natl Acad. Sci. USA*, **87**, 7160–7164.

ROCHLITZ, C.F., SCOTT, G.K., DODSON, J.M., LIU, E., DOLLBAUM, C., SMITH, H.S. & BENZ, C.C. (1989). Incidence of activating ras oncogene mutations associated with primary and metastatic human breast cancer. *Cancer Res.*, **49**, 357–360.

ROCHLITZ, C.F., HEIDE, I., DE KANT, E., BÖHMER, R., PETER, F.J., NEUHAUS, P., HUHN, D. & HERRMANN, R. (1993). Position specificity of Ki-ras oncogene mutations during the progression of colorectal carcinoma. *Oncology*, **50**, 70–76.

SANTIBANEZ-KOREF, M., BIRCH, J.M., HARTLEY, A.L., MORRIS JONES, P.H., CRAFT, A.W., EDEN, T., CROWTHER, D., KELSEY, A.M. & HARRIS, M. (1991). p53 germline mutations in Li-Fraumeni syndrome. *Lancet*, **338**, 1490–1491.

SRIVASTA, S., ZOU, Z.Q., PIROLLO, K., BLATTNER, W.A. & CHANG, E.H. (1990). Germ-line transmission of a mutated p53 gene in a cancer-prone family with Li-Fraumeni syndrome. *Nature*, **348**, 747–759.

TOGUCHIDA, J., YAMAGUCHI, T., DAYTON, S.H., BEAUCHAMP, R.L., HERRERA, G.E., ISHIZAKI, K., YAMAMURO, T., MEYERS, P.A., LITTLE, J.B., SASAKI, M.S., WEICHSELBAUM, R.R. & YANDELL, D.W. (1992). Prevalence and spectrum of germline mutations of the p53 gene among patients with sarcoma. *N. Engl. J. Med.*, **326**, 1301–1308.

TURCOT, J., DESPRES, J.-P. & ST. PIERRE, F. (1959). Malignant tumours of the central nervous system associated with familial polyposis of the colon. Report of 2 cases. *Dis. Colon Rectum*, **2**, 467–468.

VOGELSTEIN, B., FEARON, E.R., HAMILTON, S.R., KERN, S.E., PREISINGER, A.C., LEPPERT, M., NAKAMURA, Y., WHITE, R., SMITS, A.M.M. & BOS, J.L. (1988). Genetic alterations during colorectal-tumor development. *N. Engl. J. Med.*, **319**, 525–532.

VON DEIMLING, A., EIBL, R.H., OHGAKI, H., LOUIS, D.N., VON AMMON, K., PETERSEN, I., KLEIHUS, P., CHUNG, R.Y., WIESTLER, O.D. & SEIZINGER, B.R. (1992). p53 mutations are associated with 17p allelic loss in grade II and grade III astrocytoma. *Cancer Res.*, **52**, 2987–2990.